# A253 PRIMARY PROPHYLAXIS FOR SPONTANEOUS BACTERIAL PERITONITIS IN HOSPITALIZED CIRRHOTIC PATIENTS WITH LOW PROTEIN ASCITES AND RENAL DYSFUNCTION OR LIVER FAILURE: A RETROSPECTIVE REVIEW FROM A TERTIARY CENTRE IN BRITISH COLUMBIA

**DOI:** 10.1093/jcag/gwac036.253

**Published:** 2023-03-07

**Authors:** A Fetz, L Li, C Lee, L Leung

**Affiliations:** University of British Columbia, Vancouver, Canada

## Abstract

**Background:**

Spontaneous bacterial peritonitis (SBP) is a severe and often fatal infection that can occur in patients with cirrhosis and ascites. The benefits of primary prophylaxis with antibiotics for SBP have been demonstrated in patients with cirrhosis presenting with gastrointestinal (GI) bleeding; patients hospitalized for other reasons with an ascitic protein less than 10 g/L; and patients with ascitic protein less than 15 g/L with either impaired renal function (serum creatinine greater than 106 µmol/L, BUN greater than 8.9 mmol/L, or serum sodium less than or equal to 130 mEq/L) or liver failure (Child-Pugh score greater than or equal to 9 or bilirubin greater than 50 umol/L).

**Purpose:**

To evaluate the rate of primary prophylaxis in patients discharged from a tertiary care hospital with low protein ascites and impaired renal function or liver failure, and subsequent episodes of SBP, hospitalizations, or deaths.

**Method:**

A retrospective chart review at St. Paul’s Hospital in Vancouver, British Columbia, from November 2019 to August 2021 was conducted. Hospitalized patients with cirrhosis who had an ascitic protein less than 15 g/L and met criteria for either renal dysfunction or liver failure were included in the study. The rate of primary prophylaxis prescribed in eligible patients as well as the subsequent incidence of SBP, hospitalizations, or all-cause mortality were evaluated. Patients were followed up to 12 months after the index paracentesis.

**Result(s):**

A total of 279 patients with cirrhosis were hospitalized during the study period. 69 patients underwent a diagnostic paracentesis and 41 patients met the inclusion criteria for primary SBP prophylaxis. 28 patients were excluded with most common reasons being ascitic protein above 15 g/L (n=12), no documented ascitic protein concentration (n=9), or index paracentesis met the criteria for the diagnosis of SBP (n=5). Of the patients included, 37 (90.2%) did not receive primary prophylaxis. 8 of these patients (21.6%) developed subsequent SBP. 30 patients (81.1%) were hospitalized at least once in the following 12 months. 18 (48.6%) died during the follow-up period with 1 death attributed to SBP. 4 patients (9.76%) received primary prophylaxis and were prescribed either ciprofloxacin or trimethoprim/sulfamethoxazole. None of these patients developed SBP, however, 3 (75%) were hospitalized and died from other causes.

**Conclusion(s):**

The rate of primary prophylaxis for SBP in hospitalized patients with low protein ascites and impaired renal function or liver failure at our institution is low. The guarded prognosis in this subset of patients is also demonstrated. Further studies are needed to assess the root causes for the lack of primary prophylaxis given.

**Please acknowledge all funding agencies by checking the applicable boxes below:**

None

**Disclosure of Interest:**

None Declared

